# Demographics of Physician Associates (PAs) in Obstetrics and Gynecology: Where They Work and How They Compare to Other PAs

**DOI:** 10.1155/2024/3057597

**Published:** 2024-02-21

**Authors:** Melissa A. Rodriguez, Roderick S. Hooker, Kasey K. Puckett, Andrzej Kozikowski

**Affiliations:** ^1^Winnie Palmer Hospital OB/GYN Hospitalist Group, Orlando, FL, USA; ^2^Independent Researcher, Ridgefield, WA, USA; ^3^National Commission on Certification of Physician Assistants, 12000 Findley Road Suite 200, Johns Creek, GA 30097, USA

## Abstract

As of 2020, maternal and infant health in the US has worsened. At the same time, the number of health professionals available to manage female health issues is changing; the number of physicians in obstetrics and gynecology (Ob-Gyn) and midwives is decreasing, whereas the number of Ob-Gyn physician associates (PAs) is growing. We analyzed PAs practicing in the Ob-Gyn discipline, drawing on the PA Professional Profile, a database maintained by the National Commission on Certification of PAs. In 2021, there were 1,322 Ob-Gyn PAs (1.2% of all clinically active PAs). This health profession has grown by 66.9% since 2013, when only 792 PAs practiced in this specialty. As of 2021, their median age was 38, and 98.0% were female (70.1% of all PAs were female). The practice setting was between office (54.7%) and hospital (34.0%) employment, with 11.3% described as “other.” In 2021, the median annual income of Ob-Gyn PAs was $105,000. With the reduction of obstetrician-gynecologists, the relative growth of PAs in this area of medicine and surgery is a natural part of the solution to the projected obstetrical physician deficit.

## 1. Introduction

The demand for obstetrics and gynecology physician associates (PAs) is growing at a time of shortage of obstetricians and gynecologists (Ob-Gyn) [1]. From 2021 to 2030, Ob-Gyn physicians will decline by 7% (from 50,850 to 47,490). In contrast, the demand will likely expand by 4% (from 50,850 to 52,660) over the same period [2]. Despite the general shortage of physicians, available studies are yet to depict the role and responsibilities of Ob-Gyn PAs or their contribution to female health. Knowing their practice settings and geographic location is needed to understand where they practice, their role, and the services provided.

In 2021, the Bureau of Labor Statistics reported that 7,100 certified nurse-midwives (CNMs) and certified midwives (CMs) were employed (down from 11,000 in 2010) [3]. The women's health nurse practitioner's (WHNP) role overlaps with family medicine and may number 7,000 or 3.3% of their cadre [4]. Furthermore, the scope of practice differs somewhat amongst the four types of providers (Table 1). A recent study suggests that PAs, more than NPs, are more likely to be involved in obstetrical and gynecological surgery [5, 6].

In 2016, the American College of Obstetrics and Gynecology (ACOG), in collaboration with the American Academy of Physician Associates (AAPA), released a report on team-based care in the Ob-Gyn practice setting [7]. The report underscores that all health professionals must function to the full extent of their training, credentials, and expertise as part of integrated, high-functioning teams to meet the growing needs of patients.

More obstetric and gynecologic care providers are needed to increase access to high-quality care throughout the lifespan [8]. We set out to create a base of understanding regarding Ob-Gyn PAs. Their demographics and practice settings are the first step in essential information that will serve as a foundation and reference for greater exploration of this PA role and activity.

A 2023 study identified Ob-Gyn PAs as proceduralists, first assistance in surgery, and independently performing outpatient surgery. Most PAs (88.7%) provide a first-assist role in various surgeries, including open, laparoscopic, and robotic-assisted cases. Categories of surgery included cesarean section, hysterectomy, salpingo-oophorectomy, and subspecialty surgeries such as oncology and urogynecology. In the outpatient setting, Ob-Gyn PAs listed over 40 procedures ranging from biopsies of the endometrium, cervix, vagina, and vulva, as well as fetal assessment, ultrasonography, and long-acting contraceptive insertion and removals [9].

These new findings about an advanced proceduralist role suggest that Ob-Gyn PAs may complement Ob-Gyn surgeons with a growing scarcity of medical and surgical providers for female conditions [10]. To this end, identifying the number of PAs in this specialty and their characteristics is needed and serves as a springboard for the role delineation of this growing PA specialty.

Moreover, how PAs in Ob-Gyn compare to PAs practicing in the total of all other 69 medical and surgical disciplines is important for workforce planning. PAs are unique among healthcare providers in that they can switch specialties. Over half (53.5%) do so at least once during their career [11]. Knowing to what degree PAs in Ob-Gyn, relative to their colleagues in other specialties, are satisfied with their clinical positions, their burnout levels, as well as intentions to leave their current positions, can help to understand better their retention and recruitment and thus future growth of the specialty among PAs. How many PAs in Ob-Gyn are available, where they are geographically located, and how they practice relative to PAs in other specialties are crucial for policymakers to understand to make informed decisions about how to allocate resources and develop policies to ensure that there is a sufficient and qualified Ob-Gyn workforce to meet the needs of the population.

### 1.1. The Research Questions

What are the demographics and characteristics of PAs in Ob-Gyn and how do they compare to PAs practicing across all other specialties?

## 2. Methods

This study was reviewed by the Sterling Institutional Review Board (IRB# 8759). Data for this cross-sectional study were extracted from the National Commission on Certification of PAs (NCCPA). Since 2012, NCCPA has collected robust data on certified PAs' demographics, practice characteristics, and other health workforce-related information. These data are collected via the PA Professional Profile (PA Profile) housed on a secure portal on the organization's website. Additionally, NCCPA gathers administrative data such as the year of initial certification. The PA Profile was designed based on the Health Resources and Services Administration Center for Workforce Studies' minimum dataset recommendations to collect essential health workforce information [12]. PAs can respond to the optional items on various topics related to their practice. The variables in the Profile instrument are standard and have remained mostly unchanged (except for adding new questions) since its launch in 2012 [13]. NCCPA welcomes applications from researchers seeking to obtain deidentified and aggregated PA workforce data. Requests are only approved for ethical research purposes. NCCPA adheres to quality control procedures to ensure the accuracy and reliability of its data [14].

Primary inclusion criteria were that clinically active PAs provide their specialty and update their information or confirm that it was up to date within the past three years. By the end of 2021, there were 158,470 certified PAs; 133,905 responded, updated, or confirmed information in the PA Profile questionnaire (84.5%). Of the 133,905 PAs, 111,726 indicated that they work in at least one clinical position, and of these, 111,428 provided their specialty. Our analytical sample of 111,428 represents 70.3% of all certified PAs in the US. For this study, we analyzed the following three sets of health workforce variables:

- Demographics (age, gender, race, ethnicity, US region, rural-urban setting, and whether PAs speak with their patients in a language other than English).
- Practice characteristics (practice setting, years certified (a proxy for the number of years practicing as a PA), hours worked in principal position per week, number of patients seen per week, whether PAs worked in a secondary role, and participation in telemedicine).
- Other health workforce variables (intent to leave the principal position in the next 12 months, planning to retire in the next five years, job satisfaction, burnout levels, and yearly income).

Most variables for PAs practicing in Ob-Gyn had 1.0% or less missing data. Variables such as race, ethnicity, and speaking a language other than English with patients had 3.0% or fewer missing data. The highest proportions of missing responses were for burnout symptoms and job satisfaction (both 9.0%) and income (6.0%). Data were summarized using descriptive statistics. For continuous variables, means, standard deviations (SDs), medians, and interquartile ranges were computed. For categorical variables, frequencies and percentages were calculated. In bivariate analyses, we conducted chi-square tests of independence and Mann–Whitney tests to determine statistical differences between PAs practicing in Ob-Gyn versus all other specialties concerning demographics, practice characteristics, and other essential variables. Data management and analysis were performed using SPSS (version 28).

## 3. Results

**Figure 1:**
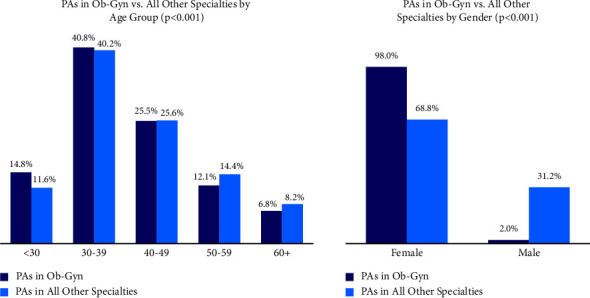
Age and gender of PAs: Ob‐Gyn vs. all other specialties.

By the end of 2021, a total of 1,322 PAs in the US self-identified as practicing in the Ob-Gyn discipline, representing 1.2% of the clinically active PA population that provided specialty information (*N* = 111,428). As seen in Figure 1, Ob-Gyn PAs were significantly more likely to be female than those practicing in other disciplines (98.0% vs. 68.8%; *p* < 0.001). The median age was slightly younger at 38 years vs. 39 years for PAs in all other specialties (*p* < 0.001). Moreover, Ob-Gyn PAs had a higher proportion of being under 30 years old while having a lower ratio in the 60 and over age group (14.8% vs. 11.6% and 6.8% vs. 8.2%; *p* < 0.001, respectively).

We detected statistically significant differences in the urban-rural setting (*p*=0.040), ethnicity (*p*=0.010), and whether PAs speak a language other than English with patients (*p* < 0.001) but not in race (*p*=0.076; Table 2). When compared to PAs in all other specialties, PAs in Ob-Gyn were more likely to work in an urban geographic setting (94.0% vs. 92.4%), identify as Hispanic/Latino (8.4% vs. 6.6%), and speak a language other than English with patients (29.9% vs. 22.6%).

**Figure 2:**
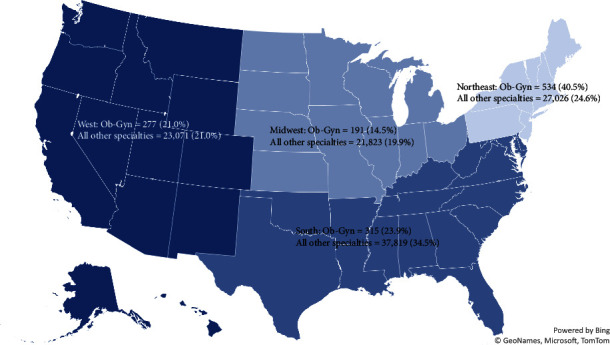
PAs in Ob‐Gyn vs. all other specialties by US region (*p* < 0.001).

Figure 2 illustrates the geographic distribution of Ob-Gyn PAs compared to PAs practicing in all other specialties, which was also statistically significant (*p*<0.001). Ob-Gyn PAs were more likely to reside in the northeast US region than all other PAs (40.5% vs. 24.6%).

Table 3 summarizes that over half (54.7%) of Ob-Gyn PAs reported being in an office-based private practice, which was significantly higher than PAs in all other specialties (37.2%; *p* < 0.001). However, a sizeable proportion indicated working in the hospital (34.0%), and 11.3% described their work setting as “other.” Almost two-thirds (64.6%) identified working full-time (31–40 hours per week). PAs in Ob-Gyn vs. those in all other specialties were slightly more likely to rely on telemedicine for patient care (36.3% vs. 33.5%; *p*=0.033). Among Ob-Gyn PAs, 87.2% said they spend fewer than 10 hours per week using telemedicine.

**Figure 3:**
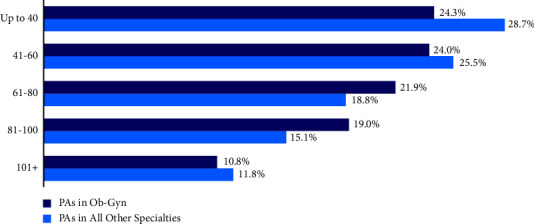
PAs in Ob‐Gyn vs. all other specialties by patients seen each week (*p* < 0.001).

Regarding the productivity of PAs, the majority (75.7%) specified seeing more than 40 patients per week (Figure 3). Moreover, we observed significant differences in the number of patients seen per week by Ob-Gyn PAs vs. PAs in all other disciplines (*p* < 0.001). Ob-Gyn PAs had a higher likelihood than PAs in other specialties to report seeing between 81–100 patients (19.0% vs. 15.1%) per week.

**Figure 4:**
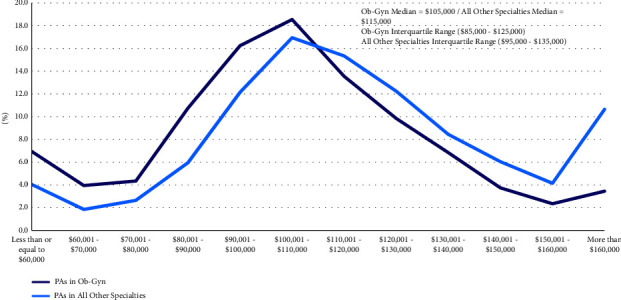
PAs in Ob‐Gyn vs. all other specialties by income (*p* < 0.001).

The median self-reported income of Ob-Gyn PAs was $105,000 in 2021 [interquartile range: $85,000–125,000], which was lower than that of PAs practicing in all other specialties ($115,000 [interquartile range: $95,000–135,000]; *p* < 0.001). However, the income bracket distribution curve for Ob-Gyn PAs was similar to that of PAs in other specialties (between 1.7% and 7.2% difference; Figure 4). The most significant difference was identified for the income bracket of more than $160,000.

Of the 1,322 PAs in Ob-Gyn in 2021, almost all (90.3%) reported being satisfied with their principal position. Moreover, their job satisfaction level was significantly higher than those practicing in all other disciplines (*p* < 0.001). Compared to PAs in other specialties, PAs in Ob-Gyn were less likely to report one or more symptoms of burnout (26.5% vs. 30.7%; *p*=0.002). Nearly all declared no intention of leaving their current employment (93.5%) or retiring within the next five years (95.8%) (Table 4).

## 4. Discussion

This is the first research study on the demographics and practice characteristics of PAs working in Ob-Gyn. As of 2021, approximately 1% of U.S. PAs practiced medicine in this field. As the PA profession has grown over the past decade, the number of PAs in Ob-Gyn has increased from 792 in 2013 to 1,322 in 2021, a doubling effect [15]. The median age of this PA cohort was 38 years. This cadre of PAs also reported high levels of weekly productivity: 80.5% worked over 30 hours per week, one-third (34.0%) practiced in a hospital setting, and almost all (98.0%) were female. The broad characteristics of these findings suggest that Ob-Gyn PAs value their role. Additionally, their income is comparable to that of PAs across all other specialties.

The American College of Gynecology projected a total of 8,800 Ob-Gyn physicians by 2020. That prediction has been realized. Furthermore, a shortfall of up to 22,000 is predicted by 2050 [6]. Projected deficits in Ob-Gyn were from microsimulation forecasts based on the growing US population and the relatively static number of residency graduates [16]. Additional factors include generational changes, working fewer hours, earlier retirement, and an increase in female physicians [6]. Overall, the physician workforce in the US is predicted to have worsening shortages by 2030 [17]. Nationally, by 2030, the Ob-Gyn number was estimated to decline by 7% [18]. Although all states have at least one county without an Ob-Gyn, the largest counties without an Ob-Gyn were in the central and mountain western regions. Over 8% of all adult women in the US, or 10.1 million, lived in counties that did not have an Ob-Gyn [6].

Changes in the female population's healthcare needs are observed during demographic shifts, and as a result, physicians per capita are decreasing [19]. Two significant observations are the increased use of long-acting contraceptives and, generally, a declining birth rate since 2000 [2]. In 2020, the number of births was 3,613,647, with a birth rate of 11.0 per 1,000 population [20]. The fertility rate is 56.0 births per 1000 women between the ages of 15 and 44. At the same time, more women visit Ob-Gyn offices for their well-woman examinations, and few are undergoing major gynecological surgeries. This may be due to minimally invasive surgeries becoming more common [20].

Additionally, care by an Ob-Gyn provider beyond the reproductive years declines as female patients shift to primary care; however, they seek gynecological care on topics such as menopause, cervical and breast cancer screening, urinary difficulties, and osteoporosis [21]. Despite a decreased birth rate, the US has seen a steady rise in maternal mortality rates. In the US in 2020, 861 women died of maternal causes; overall, the maternal mortality rate was 23.8 deaths per 100,000 live births. Alarmingly, it was 55.3 deaths per 100,000 live births for African American women, an almost threefold higher rate than for White women [22].

PAs have been employed in Ob-Gyn since the beginning of the profession in 1967. Postgraduate programs in Ob-Gyn began as early as 1972 to fill a void in female health and to treat high-risk, low-income, and underserved women [23]. In this decade, PA programs varied little in structure, with most consisting of a didactic and clinical portion of approximately 24–45 months in length and adhering to a design by a central accreditation system [24]. Two postgraduate programs in Ob-Gyn for PAs are at Arrowhead Regional Medical Center and previously at Montefiore Medical Center [25].

The utilization of PAs as obstetrical laborists has been a strategy in the northeast region of the US to adjust to concentrated areas of maternity and delivery. Dedicated laborists provide continuous coverage of laboring patients. They can improve care quality, enhance patient safety, and help offset Ob-Gyn physicians' demands. Owing to their advanced education and training, PAs have a versatile skill set in Ob-Gyn, surgery, and emergency care, making them qualified to practice in various settings [26].

In summary, the American PA movement is expanding and brings a needed corps of specialists in Ob-Gyn. This medical specialty is represented by the *Association of Physician Associates in Obstetrics and Gynecology (APAOG)*. The next step is understanding how this group collaborates and contrasts with other female health specialists.

### 4.1. Limitations

This study is composed of data from NCCPA's PA Professional Profile. The PA Profile remains the most comprehensive national collection of PA workforce data and is in a constant state of growth and improvement. However, this study has limitations. First, the PA Profile relies on self-reported health workforce data, which may contain inherent limitations. These may include measurement and nonresponse errors and social desirability biases. Not all certified PAs may have provided their specialty information, so our findings could be an underestimate of the total number of certified PAs practicing in the Ob-Gyn discipline. Although missing data were minimal for most of the variables we assessed, job satisfaction and burnout levels had the highest, with 9% missing responses. Lastly, PAs in other disciplines, such as primary care and surgery, render care for female health issues but were not explored in this study.

## 5. Conclusion

In 2021, 1,322 PAs in Ob-Gyn (1.2% of all clinically active PAs) were distributed throughout the US and working in hospitals and outpatient settings. Almost all were female (98.0%), and their median age was 38. Most worked in urban settings (94.0%), office-based private practice (54.7%), and hospitals (34.0%). Approximately 6.1% worked in rural settings. PAs practicing in Ob-Gyn were likelier to indicate being satisfied with their position and less likely to report burnout, intending to leave their position and retire than their colleagues in other specialties. These findings suggest that the Ob-Gyn specialty among PAs is expected to grow. Knowing the characteristics of PAs in Ob-Gyn is needed to understand how these health professionals respond to the dynamics of medical labor supply and demand. While this medical group has yet to be role-defined in the literature, newer information suggests that Ob-Gyn PAs represent a needed provider at the time of increasing need. This foundation of the modern PA workforce involves optimizing female health.

## Figures and Tables

**Table 1:** Current education and scope of practice for specialized women's health clinicians.

Profession	Degree	Training time	Scope of practice			
			Prenatal	Obstetrics/labor and delivery	Gynecology	Gynecological surgery
Physician (MD/DO)	Doctorate	8 years (4 years medical school + 4 years residency)	Yes	Yes	Yes	Yes
Physician associate	Master's	3 years	Yes	Yes	Yes	Yes, first assistance
Women's health nurse practitioner	Doctorate available	2-3 years	Yes	No	Yes	No
Nurse midwife	Master's	2-3 years	Yes	Yes	Yes	No

Modified from American College of Obstetricians and Gynecologists. Task force on collaborative practice. Collaboration in practice: implementing team-based care/developed under the direction of the task force on collaborative practice. District of Columbia. 2016; American Association of Nurse Practitioners. 2020 AANP national nurse practitioner sample survey. https://storage.aanp.org/www/documents/no-index/research/2020-NP-Sample-Survey-Report.pdf; Bureau of Labor Statistics, 2022.

**Table 2:** Demographic characteristics of PAs practicing in Ob-Gyn vs. all other specialties.

	PAs practicing in Ob-Gyn	PAs practicing in all other disciplines	*p* value
*Race*			
White	1,067 (83.6%)	89,315 (84.9%)	0.076
Asian	74 (5.8%)	6,343 (6.0%)	
Black/African American	61 (4.8%)	3,630 (3.4%)	
Other^*∗*^	75 (5.9%)	5,972 (5.7%)	
*Ethnicity*			
Non-Hispanic/Latino	1,178 (91.6%)	98,714 (93.4%)	0.010
Hispanic/Latino	108 (8.4%)	6,987 (6.6%)	
*Urban-rural setting*			
Urban	1,233 (94.0%)	101,256 (92.4%)	0.040
Large rural	52 (4.0%)	4,751 (4.3%)	
Isolated/small rural	27 (2.1%)	3,557 (3.2%)	
*Speaks languages other than English with patients*			
No	903 (70.1%)	82,885 (77.4%)	<0.001
Yes	385 (29.9%)	24,201 (22.6%)	

^*∗*^Other includes those who selected “other,” Native Hawaiian/Pacific Islander, American Indian or Alaska Native, and “multi-race.”

**Table 3:** Practice characteristics of PAs practicing in Ob-Gyn vs. all other specialties.

	PAs practicing in Ob-Gyn	PAs practicing in all other disciplines	*p* value
*Practice setting*			
Office-based private practice	721 (54.7%)	40,916 (37.2%)	<0.001
Hospital	448 (34.0%)	45,832 (41.7%)	
Other	149 (11.3%)	23,219 (21.1%)	
*Years certified groups*			
Up to 10	636 (48.1%)	57,050 (51.8%)	0.018
11–20	428 (32.4%)	33,948 (30.8%)	
21+	258 (19.5%)	19,108 (17.4%)	
*Years certified*			
Mean (SD)	12.8 (9.4)	12.1 (8.8)	0.079
Median (IQR)	11 (5–19)	10 (5–18)	
*Hours worked per week*			
Up to 30	257 (19.5%)	14,392 (13.1%)	<0.001
31–40	854 (64.6%)	61,927 (56.3%)	
41–50	174 (13.2%)	26,590 (24.2%)	
51+	36 (2.7%)	7,183 (6.5%)	
*Participate in telemedicine*			
No	835 (63.7%)	72,875 (66.5%)	0.033
Yes	476 (36.3%)	36,733 (33.5%)	
*Hours worked in telemedicine each week*			
<10	415 (87.2%)	27,968 (76.2%)	<0.001
10+	61 (12.8%)	8,745 (23.8%)	

**Table 4:** Other important characteristics of PAs practicing in Ob-Gyn vs. all other specialties.

	PAs practicing in Ob-Gyn	PAs practicing in all other disciplines	*p* value
*Job satisfaction*			
Not satisfied^*∗*^	117 (9.7%)	14,567 (14.9%)	<0.001
Satisfied^*∗∗*^	1,091 (90.3%)	83,442 (85.1%)	
*Burnout*			
No symptoms of burnout	885 (73.5%)	67,900 (69.3%)	0.002
One or more symptoms of burnout	319 (26.5%)	30,071 (30.7%)	
*Intend to leave principal clinical position in the next 12 months*			
No	1,231 (93.5%)	101,191 (92.2%)	0.072
Yes	85 (6.5%)	8,554 (7.8%)	
*Plan to retire in 5 years*			
No	1,253 (95.8%)	103,331 (94.6%)	0.053
Yes	55 (4.2%)	5,922 (5.4%)	

^*∗*^Not satisfied includes “neither satisfied nor dissatisfied,” “somewhat dissatisfied,” “mostly dissatisfied,” and “completely dissatisfied.” ^*∗∗*^Satisfied includes “completely satisfied,” “mostly satisfied,” and “somewhat satisfied.”

## Data Availability

NCCPA has a process that researchers can apply to obtain deidentified and aggregated PA workforce data it collects. Requests are only approved for ethical research purposes. NCCPA adheres to quality control procedures to ensure accurate and reliable data.
